# A comparative study of fluoride ingestion levels, serum thyroid hormone & TSH level derangements, dental fluorosis status among school children from endemic and non-endemic fluorosis areas

**DOI:** 10.1186/2193-1801-3-7

**Published:** 2014-01-03

**Authors:** Navneet Singh, Kanika Gupta Verma, Pradhuman Verma, Gagandeep Kaur Sidhu, Suresh Sachdeva

**Affiliations:** Department of Oral Pathology and Microbiology, Maharaja Ganga Singh Dental College and Hospital, Sri Ganganagar, Rajasthan India; Department of Pedodontics and Preventive Dentistry, Surendra Dental College and Research Institute, HH Gardens, Power house road, Sri Ganganagar, 335001 Rajasthan, India; Department of Oral Medicine and Radiology, Surendra Dental College and Research Institute, Sri Ganganagar, Rajasthan, India; Department of Oral Pathology and Microbiology, Darshan Dental College and Hospital, Udiapur, Rajasthan India

**Keywords:** Fluorosis, Water, Thyroid, Udaipur

## Abstract

The study was undertaken to determine serum/urinary fluoride status and comparison of free T4, free T3 and thyroid stimulating hormone levels of 8 to 15 years old children with and without dental fluorosis living in an endemic and non-endemic fluorosis area. A sample group of 60 male and female school children, with or without dental fluorosis, consuming fluoride-contaminated water in endemic fluoride area of Udaipur district, Rajasthan were selected through a school dental fluorosis survey. The sample of 10 children of same age and socio-economic status residing in non endemic areas who did not have dental fluorosis form controls. Fluoride determination in drinking water, urine and blood was done with Ion 85 Ion Analyzer Radiometer with Hall et al. method. The thyroid gland functional test was done by Immonu Chemiluminiscence Micropartical Assay with Bayer Centaur Autoanalyzer.

The significantly altered FT_3,_ FT_4_ and TSH hormones level in both group1A and 1B school children were noted. The serum and urine fluoride levels were found to be increased in both the groups. A significant relationship of water fluoride to urine and serum fluoride concentration was seen. The serum fluoride concentration also had significant relationship with thyroid hormone (FT3/FT4) and TSH concentrations. The testing of drinking water and body fluids for fluoride content, along with FT3, FT4, and TSH in children with dental fluorosis is desirable for recognizing underlying thyroid derangements and its impact on fluorosis.

## Introduction

Iodine Deficiency Disorders (IDD) and fluorosis are the two most prevalent endemic diseases which coexist in certain regions in India (Hetzel et al. 1990). As early as 1928, Stocks (1928) observed that children consuming well water in the village of Somerset, England exhibited both goiter and mottled enamel (dental fluorosis). Some years later, Wilson (1941) found dental fluorosis (DF) associated with goiter and cretinism among children living in areas of Punjab where fluoride was recognized geologically to be significantly high. Besides dental fluorosis and cretinism, children in endemic fluorosis areas of India often have low IQ, deaf mutism, knock-knee and bow-legs (Susheela 2003). The DF is a developmental disorder which is due to aberrant thyroid hormone metabolism. These are serious public health problems of great concern. Since fluoride is known to interfere with thyroid gland function and to cause degenerative changes in the central nervous system, impairment of brain function and abnormal development in children (Ha et al. 1989), further investigation is clearly needed, even where iodine intake is not deficient.

As it is already known that fluoride is more electronegative than iodine, it easily displaces iodine within the body, thereby affects the functioning of thyroid gland. Fluoride has been known to have shown gross as well as biochemical changes within the body of an individual which included deranged thyroid hormonal level with in the body. The production of thyroid hormones is regulated by a negative feedback mechanism, i.e., when the pituitary gland senses a drop in FT_3_ levels in circulation, it releases more TSH to stimulate the thyroid gland which in turn accelerates the production of the thyroid hormone T_4_, now considered a “pro-hormone”. The major source of circulating T_3_ is from peripheral deiodination of T_4_ and not from thyroid secretion. The enzymes which catalyze deiodination are called iodothyronine deiodinases and fluoride is known to interfere with the activity of the deiodinases.

The effect of the fluoride on tooth development is dose dependent. Their effects on various stages of tooth development, on dental tissues and on enamel in particular have been related to the interaction between fluoride ions and calcium hydroxyapatite besides the regulatory effects of thyroid secretions. To gain a better understanding of the problem, the present study was undertaken to determine the fluoride status and compare it with the free T4, free T3, and thyroid stimulating hormone (TSH) levels of children with and without dental fluorosis living in an endemic fluorosis area.

## Materials and method

The study was conducted to evaluate and correlate the effect of chronic excess fluoride intake on thyroid function among school children (8–15 years) from endemic and non-endemic fluorosis areas. The villages with high fluoride levels in the drinking water in the Udaipur district of Rajasthan, India were included as endemic areas (Group 1). The villages which were selected were Slumber, Sarada, Kalutada, Devgaun and Kejad. Group 1 included 60 male and female school children, which were equally divided into two subgroups: Group 1A (children with dental fluorosis) and Group 1B (children without dental fluorosis).

Group 2 included 10 children from Sardarpura colony of Udaipur city, a non endemic area, which was taken as a control for the study samples.

It has been reassured that the study was conducted in conformity with principles in Helsinki Declaration and others specified in the respective ethics committees of Rajasthan University. In group 1A (n = 30), 15 children were selected from areas having water fluoride level up to 2.6 ppm while the remaining 15 children were from areas having water fluoride level up to 5.1 ppm. In group 1B (n = 30), 15 children were selected from areas having water fluoride level up to 2.6 ppm while the remaining 15 children were from areas having water fluoride level up to 5.1 ppm. The group 2 children (n = 10) were of the same age range and socio economic status, residing in the non-endemic area, without exhibiting dental fluorosis and serving as the control group. Their drinking water was deemed to be “safe” (<1.0 mg F–/L) and like the sample group, these control children were also investigated for thyroid gland function.

The school children’s dental history with dentition status and Dean’s index was recorded. The six grades are known as Dean’s classification scale for dental fluorosis. They are: none (normal enamel) (the score marked for 0), suspected or questionable (0.5), very mild (1), mild (2), moderate (3), and severe(4) Statistical analysis of the prevalence of dental fluorosis was made according to the rates of DF%. The morning blood sample was collected of these children by venipuncture into the tubes on the day before and serum was separated via centrifuge, for thyroid gland function tests and fluorine estimation in serum. Serum fluoride concentration is recognized as a good indicator of fluoride exposure. The samples were stored in refrigerator at 4°C and then analyzed for the fluoride levels. The drinking water fluoride estimation was done to adjudge the endemic and non-endemic areas. The Urinary fluoride is regarded as an indicator of grade of exposure to fluorine compounds absorbed.

### Method

The samples of drinking water, urine and blood were collected in plastic bottles, blood samples were left to clot at room temperature and the serum was separated by centrifugation (Negoita et al. 2001). Fluoride ion level in body fluid was estimated by manual titration method and automatic fluoride Ion 85 Ion Analyzer and radiometer with Hall et al. in (1972) and ASTDM method. Fluoride determination in the drinking water, urine and blood serum was carried out potentiometrically with an Ion 85 Ion Analyzer and a fluoride ion specific electrode and radiometer with ASTDM method. The serum samples of children were investigated to assess FT4, FT3, and TSH hormone levels using Immuno Chemiluminiscence Microparticle Assay (ICMA) with the Bayer Centaur Autoanalyzer.

## Results

All the data was statistically analyzed using SPSS 13 software. The level of fluoride naturally ingested from drinking water and body fluids in all sample groups which is varied over a wide concentration range of fluoride content in drinking water from 0.98 to 5.5 mg F–/L (ppm), in blood serum 0.02–0.77 mg F–/L (ppm) and in urine from 0.24–8.9 mg F–/L (ppm). (Table 1). Table 2 showed the thyroid (FT_3_ & FT_4_) and TSH hormone level in body fluids for all 60 children of group 1A and 1B and 10 children of Group 2. FT3 levels were recorded highest in Group 2 with minor difference in other groups; concentration of FT_4_ levels was maximum in Group 1A, whereas TSH levels were significantly higher in Group 1B.

**Table 1:** **Levels of fluoride naturally ingested from drinking water and body fluids in different sample groups**

Parameters	Group 1A	Group 1B	Group 2	Total
**Water fluoride (WF)**	1.6–5.1 ppm	1.6–5.5 ppm	0.98–1 ppm	0.98–5.5 ppm
**Urine fluoride (UF)**	0.24–8.9 ppm	0.4–7.79 ppm	0.19–1.01 ppm	0.24–8.9 ppm
**Serum fluoride (SF)**	0.02–0. 77 ppm	0.03–0.75 ppm	0.02–0.09 ppm	0.02–0.77 ppm

**Table 2:** **Levels of thyroid hormones in all sample groups**

Parameters	Group 1A	Group 1B	Group 2	Total
**Free T**_**3**_ **(FT**_**3**_ **)**	1.1–4.39 pg/ml	1.2–4.57 pg/ml	1.90–4.13 pg/ml	1.1–4.57 pg/ml
**Free T**_**4**_ **(FT**_**4**_ **)**	0.94–1.98 ng/dL	0.8–1.7 ng/dL	0.87–1.67 ng/dL	0.8–1.98 ng/dL
**TSH**	1.41–8.46 μIU/m	1.92–10.99 μIU/m	0.96–3.54 μIU/m	0.96–10.99 μIU/m

The thyroid hormone (FT_3_, FT_4_, TSH) levels revealed that the derangements are present in 72% of cases in group 1, whereas only 10% of children were involved in group 2. 50% children in group 2 had very slightly elevated serum fluoride concentration, whereas in group 1, 98% of children were involved. The children of endemic areas revealed derangement of serum thyroid hormone and TSH levels, along with increased fluoride concentration in the body fluids. This affects the tooth development in the form of delayed eruption of teeth in the children of endemic area but not in the non-endemic area (Table 3).

**Table 3:** **Derangement in Thyroid hormone (FT**_**3**_ **, FT**_**4**_ **, TSH) levels and serum fluoride levels in children of different groups**

Group	No. of cases with Derangement in Thyroid hormone (FT_3_, FT_4_, TSH) level	No. of children with abnormal serum fluoride level	No. of children with delayed eruption
**Group1A (n = 30)**	23 (77%)	29 (97%)	17 (57%)
**Group1B (n = 30)**	20 (67%)	30 (100%)	15 (50%)
**Group 2 (n = 10)**	1 (10%)	5 (50%)	0 (0%)

Table 4 indicates the correlation of various parameters with different groups. A significant correlation was found with values of TSH in different groups; whereas FT_3_, FT_4_ levels were insignificantly correlated. Level of water, serum and urine fluoride was showing highly significant correlation with both group 1 & 2.

**Table 4:** **Correlation of various parameters with Group 1 and Group 2**

Parameters	Group	n	Mean	Std. Deviation (S.D)	***t***-test	df	p-value*
FT_3_	1	60	3.0758	1.10440	1.589	68	0.117
	2	10	2.5000	0.71146			
FT_4_	1	60	1.1982	0.21692	0.259	68	0.796
	2	10	1.1790	0.21845			
TSH	1	60	3.7062	1.93841	1.940	68	0.057*
	2	10	2.4960	0.75167			
WF	1	60	2.685	1.1970	4.453	68	0.000*
	2	10	0.989	0.0099			
UF	1	60	2.6518	2.04664	3.058	68	0.003*
	2	10	0.6582	0.25726			
SF	1	60	0.1863	0.15833	2.878	68	0.005*
	2	10	0.0410	0.02807			

*Correlation is significant at the 0.05 level (2-tailed *t*-test).

Dean’s Fluorosis index was used to grade level of fluorosis in children of Group 1A. The results showed mild fluorosis in 13% children, moderate fluorosis in 67% children and severe fluorosis in 20% children, suggesting that the influence in endemic fluoride area on tooth formation is highly significant & the least effect is also so much that it causes moderate dental fluorosis as a consequence of fluoride toxicity (Table 5).

**Table 5:** **Children of group1A with Dean’s grades of fluorosis**

Dean’s fluorosis index grades	Percentage of children in Group 1A
0	0%
1	0%
2	13%
3	67%
4	20%

Table 6 showed the correlation of fluoride content in body fluids and their effect on thyroid hormones within group 1 (fluoride endemic area). Water fluoride level has very highly significant correlation with serum fluoride level, urine fluoride level and FT_3_ hormone level, whereas a significant correlation was found with FT_4_ level. Serum fluoride level has a highly significant correlation with FT_3_ and TSH. FT_3_ has highly significant correlation with TSH and significant correlation with FT_4_ hormone level.

**Table 6:** **Correlation analysis between fluoride content in body fluids and their effect FT3, FT4, TSH within Group 1**

Parameters	Spearman rho analysis	FT_3_	FT_4_	TSH	WF	UF	SF
FT_3_	'r’	1	-0.169	-0.252	-0.711	-0.388	-0.400
	p-value	-	0.196	0.052*	0.000**	0.002**	0.002**
FT_4_	'r’	-0.169	1	-0.079	0.196	0.119	0.119
	p-value	0.196	-	0.547	0.134	0.365	0.366
TSH	'r’	-0.252	-0.079	1	0.151	0.079	0.552
	p-value	0.052*	0.547	-	0.250	0.550	0.000**
WF	'r’	-0.711	0.196	0.151	1	0.690	0.529
	p-value	0.000**	0.134	0.250	-	0.000**	0.000**
UF	'r’	-0.388	0.119	0.079	0.690	1	0.525
	p-value	0.002**	0.365	0.550	0.000**	-	0.000**
SF	'r’	-0.400	0.119	0.552	0.529	0.525	1
	p-value	0.002**	0.366	0.000**	0.000**	0.000**	-

^*^Correlation is significant at 0.05 levels (2 tailed), **Correlation is highly significant below 0.01 levels correlation coefficient(r).

## Discussion

Fluoride and iodine are both halogens. The fluoride, the negative ion of the element fluorine easily displaces iodine in the body because it is much lighter and therefore more reactive. In fact the activity of any one of the halogens is inversely proportion to its atomic weight. In other words, one halogen can displace another one of a higher atomic weight but cannot displace one of lower weight thereby, results fluoride- thyroid-iodine antagonism which in turn lead to interference with iodine uptake. The fluoride is a universal G-protein activator/inhibitor. The stimulation of certain G-proteins occurs due to the toxic effects of fluoride, which has the effects of switching off the uptake into the cell of the active thyroid hormone. The thyroid control mechanism is compromised. The TSH output from pituitary gland is inhibited by fluoride, thus reducing thyroid output from thyroid glands. Fluoride competes for the receptor sites on the thyroid gland which respond to TSH; so that this hormone reaches the thyroid gland and so fewer hormone is manufactured (Wilson and DeEds 1940; Susheela et al. 2005).

The enzymes which catalyze deiodination are called iodothyronine deiodinases, of which three have been identified as D_1_, D_2_, and D_3_ (Visser and Peeters 2012). D_1_ activity is responsible for conversion of T4 to T3 in peripheral tissues, particularly in the liver and is reflected in plasma T3 levels. D_1_ activity has both outer ring deiodination as well as inner ring deiodination activity. Among the three deiodinases, D_1_ is expressed in thyroid gland besides the liver and kidney. D_2_ is found in brain, pituitary gland, and skeletal muscle and D_3_ is highly expressed in brain, placenta and fetal tissues. The present study has focused on thyroid hormones rather than the deiodinases, fluoride is known to interfere with the activity of the deiodinases.

In the present study, we found statistically significant relationship between water fluoride-urine fluoride and water fluoride-serum fluoride. Similar relationships were also observed by Rathee et al. in (2004). In another study conducted by Xiang et al. in (2009), the significant positive relation between serum fluoride and drinking-water fluoride was found. In our study, we found significant relationship between serum fluoride and TSH, serum fluoride and FT_3_/FT_4_, TSH and FT_3_/FT_4_. Similar significant relationship was also found by Susheela et al. (2005) and Wu et al. (2008). We observed that high fluoride exposure can cause functional abnormalities of thyroid and different degrees of dental fluorosis could be observed with significant deviation in the serum thyroid hormone levels, as cited by Xiang et al. (2009).

When serum FT_3_, FT_4_ and TSH of group 1 and group 2 of our study were compared we found significant difference between the two groups in serum TSH; FT_3_-FT_4_; TSH-FT_3_; TSH-FT_4_. The serum TSH, TSH-FT_3_ and TSH-FT_4_ were significantly higher unlike the results of study in rats in which contrary value of FT_3_/FT_4_ were found (Brtko et al. 1993).

In view of serum and urinary fluoride levels in the Group 2 in the present study, wherein the likelihood of fluoride ingestion from food and other sources is apparent. Hence, it is evident that analysis of fluoride in body fluids besides drinking water is highly relevant and necessary for understanding potential health implications.

The chronic over exposure of fluoride in drinking water causes growth disturbances particularly evident in adolescence and they result in thyroid dysfunction as studied by various authors, for the same reasons we also see dentition disturbances like dental fluorosis (Smith and Tafforeau 2008; Villa et al. 2010). Hence, it is evident that the analysis of fluoride in body fluids besides drinking water is highly relevant and necessary for understanding potential health implications (Martins et al. 2011). The results of the present investigation indicate that improvement in the health of the children would likely be achieved if management strategies incorporate emerging knowledge to address fluoride toxicity in the individuals, even if residing in non endemic fluoride areas.

## Conclusion

The results of this study question the validity of the fluoridation of drinking water, milk, fruit juices, and salt by public health authorities and also the step taken to prevent ill effects of excess fluorine and iodine deficiencies in endemic fluorosis areas. The children with dental fluorosis living in endemic fluorosis areas may not have a frank thyroid disease due to excessive fluorine consumption but they do show thyroid disease leading to many health effect hence they require special care and attention.
